# Effects of distance on detectability of Arctic waterfowl using double‐observer sampling during helicopter surveys

**DOI:** 10.1002/ece3.4824

**Published:** 2019-02-05

**Authors:** Ray T. Alisauskas, Paul B. Conn

**Affiliations:** ^1^ Environment and Climate Change Canada Prairie and Northern Wildlife Research Centre Saskatoon Saskatchewan Canada; ^2^ Marine Mammal Laboratory, Alaska Fisheries Science Center National Marine Fisheries Service, NOAA Seattle Washington

**Keywords:** aerial survey, detection probability, distance sampling, mark–recapture

## Abstract

Aerial survey is an important, widely employed approach for estimating free‐ranging wildlife over large or inaccessible study areas. We studied how a distance covariate influenced probability of double‐observer detections for birds counted during a helicopter survey in Canada’s central Arctic. Two observers, one behind the other but visually obscured from each other, counted birds in an incompletely shared field of view to a distance of 200 m. Each observer assigned detections to one of five 40‐m distance bins, guided by semi‐transparent marks on aircraft windows. Detections were recorded with distance bin, taxonomic group, wing‐flapping behavior, and group size. We compared two general model‐based estimation approaches pertinent to sampling wildlife under such situations. One was based on double‐observer methods without distance information, that provide sampling analogous to that required for mark–recapture (MR) estimation of detection probability, $\hat{p}$, and group abundance, $\hat{G}$, along a fixed‐width strip transect. The other method incorporated double‐observer MR with a categorical distance covariate (MRD). A priori, we were concerned that estimators from MR models were compromised by heterogeneity in $\hat{p}$ due to un‐modeled distance information; that is, more distant birds are less likely to be detected by both observers, with the predicted effect that $\hat{p}$ would be biased high, and $\hat{G}$ biased low. We found that, despite increased complexity, MRD models (ΔAICc range: 0–16) fit data far better than MR models (ΔAICc range: 204–258). However, contrary to expectation, the more naïve MR estimators of $\hat{p}$ were biased low in all cases, but only by 2%–5% in most cases. We suspect that this apparently anomalous finding was the result of specific limitations to, and trade‐offs in, visibility by observers on the survey platform used. While MR models provided acceptable point estimates of group abundance, their far higher stranded errors (0%–40%) compared to MRD estimates would compromise ability to detect temporal or spatial differences in abundance. Given improved precision of MRD models relative to MR models, and the possibility of bias when using MR methods from other survey platforms, we recommend avian ecologists use MRD protocols and estimation procedures when surveying Arctic bird populations.

## 1 INTRODUCTION

Ecologists typically consider a change in animal abundance as a metric of population health (Nichols & Hines, 2002). Use of aircraft for counting animals permits observers to draw inference about abundance over large areas that may be difficult to access otherwise (Seber, 1982:454). However, inferences drawn from counts of wildlife populations about abundance are often complicated by incomplete detection of animals: not all detectable animals present in the surveyed area are counted. As such, substantial effort has been devoted to developing survey protocols and methods of correction for detection bias (also termed—visibility bias) in aerial surveys (Pollock & Kendall, 1987). In North America, aerial surveys of waterfowl have become an integral tool for population estimation and are guided largely by standardized protocols developed by the US Fish and Wildlife Service and Canadian Wildlife Service (1987). For example, a long‐term operational survey for duck abundance has been conducted over the prairies of the interior of the continent each May since 1955 (Smith, 1995); ducks are identified by species and counted during aerial surveys of 400 m fixed‐width transects divided into ~29 km segments from aircraft flying ~50 m above ground level at 145–170 km/hr. A subsample of these segments are surveyed by ground crews that also count ducks so that a visual correction factor (VCF, i.e., the reciprocal of detection probability) can be computed to adjust all aerial counts to account for incomplete detection from the air. Other surveys, particularly in the Arctic or heavily forested habitats, use the same protocol for aerial counts but without ground counts due to remoteness and associated logistic constraints that impede access by ground observers. In such cases, count data from a single survey platform can be used to estimate detection probability (Koneff, Royle, Otto, Wortham, & Bidwell, 2008).

Historically, single platform methods were broadly classified as one of two types: (a) those that employ multiple observer protocols (e.g., Caughley & Grice, 1982; Cook & Jacobson, 1979; Grier, Gerrard, Hamilton, & Gray, 1981; Koneff et al., 2008), or (b) those that require only a single observer who records the perpendicular distance of detected groups of animals to the transect line (i.e., distance sampling, DS; Buckland et al., 2001; Burnham, Anderson, & Laake, 1980). Double‐observer methods are analogous to mark–recapture (MR) methods and exploit matched pairs of detections and non‐detections from two observers to estimate detection probabilities using, for example, a Lincoln‐Petersen estimator (Alpizar‐Jara & Pollock, 1996; Seber, 1982). Development of Horvitz–Thompson type estimators (Alho, 1990; Huggins, 1989, 1991) for mark–recapture data are a marked improvement over simple Lincoln‐Petersen estimators in that they allow investigators to control for variation in detection probabilities as a function of recorded covariates (e.g., distance, group size, species). Regardless, crucial assumptions of MR models are that observers independently detect animals and that detection probabilities are homogeneous for animals with the same detection covariates. These assumptions may be violated during aerial surveys where each observer has a shared field of view, and groups of animals that are visually distinct may be more likely to be detected by both observers than inconspicuous animals. For example, probability of detection of a group of organisms by both observers within fixed‐width strip transects may vary with distance from the survey platform (Laake & Borchers, 2004). Not accounting for such sources of detection heterogeneity within estimation is well known to lead to negatively biased abundance estimates (Seber, 1982).

By contrast, DS procedures are insensitive to moderate heterogeneity in detection probability owing to a “pooling robustness” property (Burnham et al., 2004). In the context of aerial surveys, the main weakness of conventional DS procedures is the assumption of perfect detection on the transect line (or, if distances are binned, the detection bin closest to the aircraft). This is untenable in many aerial surveys owing to the altitude of the aircraft and the complex and imperfect nature of the visual detection process. Several approaches exist to improve estimates of abundance by using distance data to reduce heterogeneity in detection. In the first, we can simply use recorded distances to animals as covariates within MR estimation (an approach we term MRD, for mark–recapture using categorical or binned distance). In the second, joint likelihoods can be specified for the mark–recapture data (detections/non‐detections) and for the distribution of observed distances. This approach is known as mark–recapture distance sampling (MRDS; Borchers, Laake, Southwell, & Paxton, 2006; Borchers, Zucchini, & Fewster, 1998; Buckland, Laake, & Borchers, 2010; Burt, Borchers, Jenkins, & Marques, 2014; Laake & Borchers, 2004). When possible to implement, MRDS is preferable because more assumptions about individual heterogeneity can be addressed. However, several features of our data set (notably, responsive movement of some animals away from the aircraft) made MRDS estimation challenging to implement. For purposes of this paper, we only provide direct comparisons of MR to MRD; we revisit this choice in the Discussion, where we compare MRD to MRDS. We note that MRD is equivalent to Burt et al.’s (2014) “MR FI” model, which uses distance as a covariate affecting detectability in MR. The only substantive difference is that MRD conditions on categorical distance data (i.e., distance is included as a factor) while the MR FI model conditions on continuous distance. However, unlike any of the Burt et al. (2014) “MRDS” models, the distribution of observed distances is never explicitly modeled (e.g., to estimate the shape of the detection function).

Koneff et al. (2008) applied the double‐observer MR approach to waterfowl counts made from fixed‐width strip transects in forested areas of eastern North America and found that detection probability of ducks was related to individual observers, aircraft type, position of observers within aircraft, and waterfowl group size. They suggested that MR methods were tractable in operational surveys; however, they also presumed that an additional covariate of aircraft distance from detections was important for reducing potential bias associated with unaccounted for heterogeneity in detection probability. However, these authors concluded that it was not operationally tractable to record perpendicular distances to animal groups from fixed‐wing aircraft, data that are needed to apply MRD or MRDS estimation.

Past helicopter surveys have also used double‐observer approaches to estimate detection probability of Arctic waterfowl (Hines & Kay, 2006; Hines, Wiebe Robertston, Kay, & Westover, 2006) applied to counts made within 200 m of aircraft. In this paper, we address the recommendation by Koneff et al. (2008) to focus on the incorporation of distance data in aerial waterfowl surveys, while accounting for effects of other covariates including bird species, group size, whether birds were flying or not, and observer position in a helicopter. Specifically, we explore the feasibility of using MRD compared to MR methods to estimate abundance or density of select species of waterfowl and other wildlife from a helicopter over Arctic habitats. In particular, we predicted that a disregard of distance as a source of heterogeneity in detection probability would result in overestimates of detection probability and underestimates of abundance, compared to an MRD approach that better accounts for detection heterogeneity.

## 2 METHODS

### 2.1 Field methods

**Figure 1:**
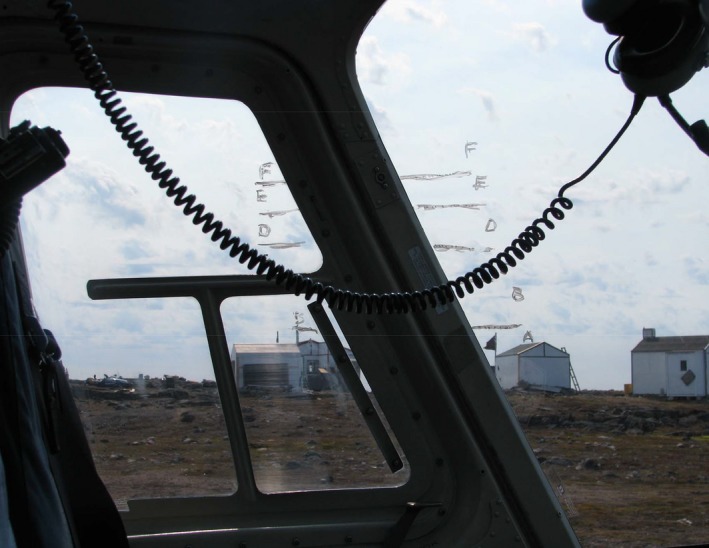
Example of distance classes marked nearest the left front seat of a Bell 206 Long Ranger helicopter

From 17 to 20 June, 2014, we used a double‐observer approach while also recording distance to detections of wildlife observations in the Queen Maud Gulf Migratory Bird Sanctuary. Observations were made from a Bell 206 Long Ranger by a forward observer in the left front seat and a rear observer in the left back seat behind the forward observer. Before any observations, each observer used a water‐soluble marker to mark semi‐transparent lines on their respective helicopter windows corresponding to distance classes of 1 = 0–40 m, 2 = 40–80 m, 3 = 80–120 m, 4 = 120–160 m, 5 = 160–200 m, and 6 > 200 m (Figure 1). Hypotenuses of triangles with a common vertical side of 50 m, but horizontal distances of 40, 80, 120, 160, and 200 m, are 64, 94, 130, 168, and 206 m, respectively. So, as the helicopter flew 50 m above ground level on this training session, each observer took a sighting to the ground with a range finder and marked a line collinear with the range finder and the appropriate hypotenuse distances to mark lines on the window with a grease pencil that represented binned perpendicular distances of 40 m intervals.

During observations, the helicopter traveled between 100 and 180 km/hr ground speed, at 50 m above ground level, consistent with US Fish and Wildlife Service and Canadian Wildlife Service (1987) protocols. Height above ground level was verified occasionally by the front observer using a range finder to determine vertical distance from ground, visible vertically through the left chin bubble of the aircraft, and communicated to the pilot whether an adjustment was required. Both observers were able to communicate via the aircraft intercom, although they were not visible to one another. Both observers recorded detections of wildlife by species, group size, whether birds were flapping their wings (usually flying, but occasionally running or stationary on the ground) or not, and class of distance perpendicular to the direction of travel by the helicopter (see above). Flapping behavior of birds was recorded because it was assumed that detection of flying groups could have been higher than of groups that were immobile and on the ground.

The forward observer did not communicate his observations to the rear observer. However, the rear observer communicated his observations via intercom to the forward observer, who recorded all detections made by either observer. If a detection of the same animal group was made by both observers, invariably the forward observer recorded his detection before the rear observer communicated his detection to the forward observer. If the rear observer detected an animal, but the forward observer did not, then this was recorded by the forward observer as such. Thus, observations by each observer were made independently of one another. An example of communication by the rear observer to the forward observer is “2 white‐fronts flying Charlie,” representing a detection in the third distance bin, 80–120 m from the helicopter (see below). The time interval between detections of different groups was sufficiently long that chances for detections of different groups by observers being erroneously treated as the same detection was very unlikely. In most cases, distance classes of the same detections were the same, although occasionally flying birds would move from one distance class where the forward observer detected the group of animals in a different (usually closer) class by the time the rear observer detected this same group; as well, there were also some distance bin mismatches between observers for bird groups that were not flapping their wings, suggesting a small amount of measurement error (Conn & Alisauskas, 2018).

### 2.2 Analysis methods

#### 2.2.1 Data formatting

Our interest in this paper was to assess hypothetical bias in estimates from MR models relative to MRD models. Such models usually assume that covariates recorded by both observers (e.g., distance) are the same, so we needed to reconcile mismatching distance data prior to analysis (see Discussion for description of an alternative approach). Let *y_io_* be a binary indicator for whether or not observer *o* detects waterfowl group *i*, and *d_io_* denote the distance bin recorded by observer *o* to group *i*. We included detection histories in the analyses if (a) *y*_i1_ = 1 and *d*_i1 _∈{1,2,3,4,5} (i.e., observer 1 detects the animal in distance bins 1–5), or (b) *y*_i1_ = 0, *y*_i2_ = 1, and *d*_i2_∈{1,2,3,4,5} (i.e., observer 1 misses the animal but observer 2 detects it in bins 1–5). The distance used for waterfowl group *i* was set to *d_i_*_ _= *y_i_*_1_ *d_i_*_1 _+ (1 − *y_i_*_1_)*d_i_*_2_ (i.e., giving preference to the first observer's distance determination). These protocols were an attempt to reduce possible bias associated with responsive movement. For instance, the original position and whether or not a group was within the strip width (0–200 m) was presumably more reliable when made by the first observer since birds would have had less opportunity to move away from the aircraft.

#### 2.2.2 Modeling double‐observer detections

We fit several MR and MRD models to double‐observer encounter histories that were a function of different combinations of predictive covariates: group size (linear effect), species (categorical, nine levels), flying/not flying (categorical, two levels), distance bin (categorical, five levels), and observer (categorical, two levels). All models that included distance bin and observer effects also included their interaction. Note that our use of categorical distance bins permitted greater modeling flexibility, although at the cost of an increased number of parameters to estimate, compared to an approach where distance is modeled as a continuous effect.

Different models corresponded to different ways that data might be analyzed under different survey protocols. For example, the MR analyses without distance covariates could be fitted with data from fixed‐width strip transects where observers do not record perpendicular distance to an object (e.g., Hines & Kay, 2006; Hines et al., 2006; US Fish & Wildlife Service & Canadian Wildlife Service, 1987). We fitted a total of 10 models to MR detection histories (Table 1).

**Table 1:** Mark–recapture models accounting for distance (MRD) versus mark–recapture models without distance data (MR) fit to double‐observer detections of waterfowl and other wildlife detected in the Arctic during aerial survey with a helicopter

Type	Model	*k*	Log*L*	AICc	ΔAICc
**MRD**	Species + Group + Fly + Observer*Distance {MRD1}	**20**	**−1024.5**	**2089.3**	**0.0**
MRD	Species + Group + Fly*Observer*Distance	28	−1017.3	2091.3	2.0
MRD	Species + Group + Observer*Distance	19	−1029.8	2098.0	8.7
MRD	Group + Fly + Observer*Distance	12	−1040.8	2105.7	16.4
MR	Species + Group + Fly + Observer	12	−1134.6	2293.4	204.1
**MR**	**Species + Group + Observer {MR1}**	**11**	**−1138.5**	**2299.1**	**209.8**
MR	Group + Observer + Fly	4	−1150.7	2309.4	220.1
MR	Species + Group	10	−1145.3	2310.7	221.4
MR	Species	9	−1151.6	2321.2	231.9
MR	Null	1	−1172.6	2347.3	258.0

Notes

Models varied by inclusion of different combinations of predictor covariates, and whether terms were additive (separated with a “+”) or were interactive (separated with a “*”) on the logit scale. Predictor covariates were Species (categorical), Group (continuous), Fly (binary; wings not flapping = 0, wings flapping = 1), Observer (binary; front seat vs. rear seat of helicopter), and Distance (categorical; 5, 40 m distance bins). We present the number of parameters (*k*), log likelihood (Log*L*), and small sample AICc scores for each fitted model (Burnham & Anderson, 2002). We highlight (in bold) the best MRD model **{MRD1}** and the MR model **{MR1**} most appropriate for estimation of detection and abundance.

We used the Huggins–Alho closed‐captures model (Alho, 1990; Huggins, 1989, 1991) to model group detection by each observer, such that

$$y_{\mathrm{io}}∼\text{Bernoulli}\left(p_{\mathrm{io}}/p_{i}^{*}\right),\;\text{where}$$

$$\text{logit}(p_{\mathrm{io}})=X\beta ,\;\text{and}$$

$$p_{i}^{*}=1-\left(1-p_{i1}\right)\;\left(1-p_{i2}\right)$$

Here, **β** denotes a column vector of logit‐linear regression parameters and **X **denotes an associated design matrix (see e.g., Draper & Smith, 1998). Under this framework, maximum likelihood is used to obtain estimates of the regression parameters, **β**. Estimates of the number of waterfowl groups (*G*_s_), and total abundance of species *s *(*N*_s_) can then be derived as.

$${\hat{G}}_{\text{s}}=\sum_{i\in \Omega_{\text{s}}}1/{\hat{p}}_{i}^{*},\;\text{and}$$

$${\hat{N}}_{\text{s}}=\sum_{i\in \Omega_{\text{s}}}g_{i}/{\hat{p}}_{i}^{*},\;\text{respectively}$$

where Ω_s_ is the set of waterfowl groups detected by at least one observer that are assigned to species *s*, and *g_i_* is the number of birds in the *i*th waterfowl group. Since our main focus here was to identify factors that affected detection, we only report estimates of ${\hat{G}}_{\text{s}}$ here.

We implemented the Huggins–Alho procedure in Program MARK (White & Burnham, 1999) via the RMark package (Laake, 2013) in the R computing environment (R Core Team, 2016). We limited observations to species categories for which we had ≥20 detection histories: Cackling goose (*Branta hutchinsii*), king eider (*Somateria spectabilis*), long‐tailed duck (*Clangula hyemalis*), northern pintail (*Anas acuta*), rock and willow ptarmigan (*Lagopus* spp.), sandhill crane (*Grus canadensis*), tundra swan (*Cygnus columbianus*), loon (*Gavia* spp.), and white‐fronted goose (*Anser albifrons*). Each species or genus was modeled using a different “group” specification within the same analysis in order to increase precision of estimates by sharing information about detection probability across taxonomic groupings (see e.g., Conn, Arthur, Bailey, & Singleton, 2006). We used Akaike's information criterion (AIC; Akaike, 1974; Burnham & Anderson, 2002) to select among models.

We assessed bias of estimators from MR models that disregard distance (i.e., those that treat data as though collected on 200 m fixed‐width strip transects), against the highest‐ranked AIC model from our candidate set (which turned out to be an MRD model). We calculated anticipated relative bias of MR estimates, $\hat{\theta }$, as

$$\text{Relative bias =}\;100\cdot \left(\frac{{\hat{\theta }}^{\text{MR}}-{\hat{\theta }}^{\text{MRD}}}{{\hat{\theta }}^{\text{MRD}}}\right)$$

## 3 RESULTS

**Figure 2:**
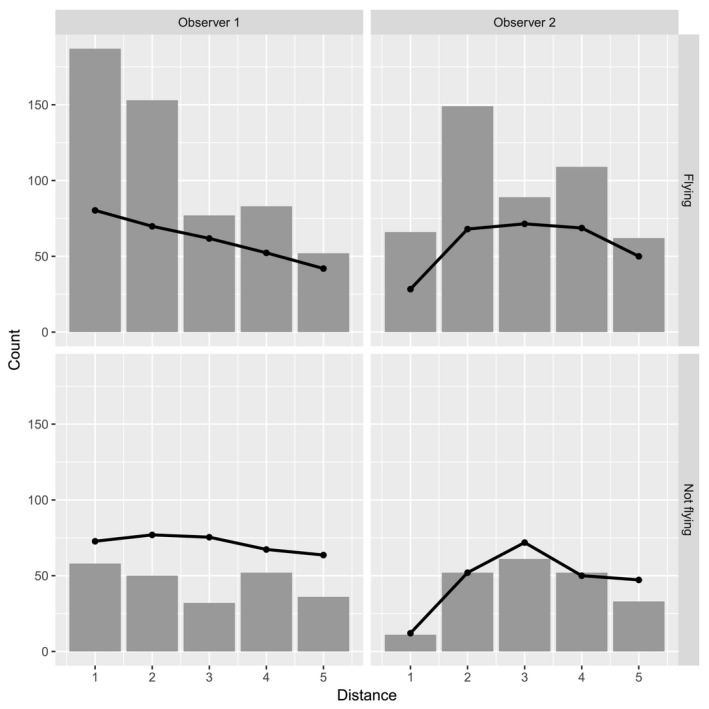
Number of waterfowl detections (bars; pooled by species) for mark–recapture distance data obtained from a Bell 206 Long Ranger helicopter, together with empirical conditional detection probabilities (plotted as a percentage; points with lines). Observations are stratified by observer (1 = front seat, 2 = back seat), and whether waterfowl were flying or not. Empirical detection probabilities were conditional on detection by the other observer. For example, points in the first column of plots were determined by using detections and distances for observer 2 as trials for observer 1

Of 1,246 detections made by either observer, 427 were made by both observers, 432 by the front observer only, and 377 by the rear observer only. The number of observations made by the front observer versus rear observer was 251 versus 50 in bin 1 (0–40 m), 209 versus 210 in bin 2 (40–80 m), 115 versus 181 in bin 3 (80–120 m), 138 versus 184 in bin 4 (120–160 m), 93 versus 105 in bin 5 (160–200 m), and 53 versus 74 beyond 200 m, respectively. Notably, detection fell off as a function of distance for both observers but was depressed for the rear observer closer to the transect line because of visual obstruction to field of view (Figure 2). This was caused by the float of the helicopter, used for buoyancy when landing on water. The front observer had a less obstructed view of bin1.

Analyses of double‐observer detection histories within a Huggins–Alho analysis suggested an influence on detection probability by all covariates considered (Table 1). In particular, MRD models (ΔAICc range: 0–16, Table 2) were far superior to MR models without distance (ΔAICc range: 204–258, Table 2). Detection probabilities varied substantially by species, observer, distance from aircraft, whether birds exhibited wing‐flapping, and group size (Table 3). Detection probability declined with distance for the front seat observer, but was gamma‐shaped (unimodal) for the rear‐seat observer, whose field of view was partially obstructed by the left helicopter float (Figure 2). Overall, detection probability was higher from the front seat than the back, was higher for flying waterfowl than non‐flying waterfowl, and increased as a function of group size, as anticipated (Table 3). Most of the species effects overlapped, but it appeared that tundra swans had a higher, although imperfect, rate of detection while loons, long‐tailed ducks, and ptarmigan had lower detection probabilities than the other species.

**Table 2:** Logit‐linear covariate effects and *SE* for the highest‐ranked AIC mark–recapture model {MRD1} from Table 1

Effect	Effect size (*SE*)
Intercept	0.50 (0.34)
Group size	0.08 (0.02)
Flying	0.59 (0.18)
Species‐KIEI	0.00 (0.24)
Species‐LTDU	−0.41 (0.25)
Species‐NOPI	0.35 (0.37)
Species‐ROPT	−0.36 (0.40)
Species‐SACR	0.27 (0.29)
Species‐TUSW	1.15 (0.32)
Species‐Loons	−1.02 (0.51)
Species‐WFGO	0.20 (0.19)
Observer 2	−2.39 (0.25)
Observer 1:Distance 2	−0.56 (0.32)
Observer 2:Distance 2	1.80 (0.21)
Observer 1:Distance 3	−1.05 (0.33)
Observer 2:Distance 3	2.18 (0.27)
Observer 1:Distance 4	−1.11 (0.32)
Observer 2:Distance 4	1.68 (0.24)
Observer 1:Distance 5	−1.42 (0.35)
Observer 2:Distance 5	1.11 (0.27)

Notes

The intercept corresponds to detection of non‐flying Canada geese by observer 1 in distance bin 1, and the “Observer 2” effect is specific to detection bin 1 viewed rear seat of a Bell 206 Long Ranger helicopter with floaths (i.e., with obstructed view).

**Table 3:** Estimates of unconditional detection probability, ${\hat{p}}^{*}$, and number of groups, $\hat{G}$, for each of nine avian species in the Arctic region covered by aerial surveys for different models, together with standard error

Species	${\hat{p}}^{*}$ (*SE*)		$\hat{G}$ (*SE*)		Relative bias (%)		
	Model{MRD1}	Model{MR1}	Model{MRD1}	Model {MR1}	${\hat{p}}^{*}$	$\hat{G}$	*SE* ($\hat{G}$)
${\hat{G}}_{\text{CAGO}}$	0.80 (0.03)	0.78 (0.03)	345 (16)	357 (17)	−3	3	6
${\hat{G}}_{\text{KIEI}}$	0.80 (0.04)	0.78 (0.04)	154 (11)	158 (11)	−2	3	0
${\hat{G}}_{\text{LTDU}}$	0.70 (0.06)	0.65 (0.06)	207 (21)	218 (23)	−7	5	10
${\hat{G}}_{\text{NOPI}}$	0.87 (0.06)	0.83 (0.07)	41 (3)	43 (4)	−4	5	33
${\hat{G}}_{\text{ROPT}}$	0.71 (0.10)	0.68 (0.10)	59 (11)	60 (11)	−4	2	0
${\hat{G}}_{\text{SACR}}$	0.85 (0.05)	0.84 (0.05)	81 (5)	85 (7)	−2	5	40
${\hat{G}}_{\text{TUSW}}$	0.96 (0.02)	0.91 (0.03)	65 (4)	66 (4)	−5	2	0
${\hat{G}}_{\text{LOON}}$	0.52 (0.15)	0.37 (0.12)	116 (39)	134 (48)	−29	16	23
${\hat{G}}_{\text{WFGO}}$	0.84 (0.03)	0.85 (0.02)	321 (10)	329 (12)	1	2	20

Notes

Estimates are from the highest‐ranked mark–recapture estimators accounting for distance (MRD1) and the highest‐ranked MR model that did not account for distance (MR1). Note that we present estimates of ${\hat{p}}^{*}$ at mean values of detection covariates. Based on results in Table 1, we posit that Model {MRD1} was the closest approximation of truth, against which relative bias was evaluated.

We expected, a priori, that failure to account for distance should have resulted in overestimation of detection probability and underestimated numbers of bird groups present on transects. Instead, the opposite tendency occurred: Failure to account for distance resulted in number of groups being consistently overestimated for all species but only moderately so (2%–5%), except for loons (16%). Failure to account for distance resulted in much greater species variation in its effect on estimates of standard errors for abundances, with either no difference for 3 species, or consistently much greater uncertainty (6%–40%) than if distance was included as a covariate.

## 4 DISCUSSION

We addressed potential bias in double‐observer aerial surveys owing to detection heterogeneity induced by animal groups observed at different distances from the aircraft. Such heterogeneity is known to cause negative bias in mark–recapture abundance estimators (Seber, 1982), which is potentially problematic for aerial strip transect surveys as commonly implemented in North American waterfowl surveys. However, despite clear potential for such bias, our abundance estimates from models that accounted for distance effects {MRD} on observations from a helicopter were remarkably similar to estimates from models that ignored those distance covariates {MR}. However, precision of some estimates was reduced considerably in our specific application. We suspect that reasonable concordance between MR and MRD estimates was related to trade‐offs in visibility issues associated with the particular configuration of the aircraft used as our survey platform. In particular, the presence of helicopter floats obviously obstructed the rear‐seat observer's field of view. Although detections by the rear observer for the closest distance bin were considerably fewer than by the forward observer's unobstructed view, the rear observer appeared to compensate for this difference by focusing greater attention on distance bins 3, 4, and 5, particularly detecting more flying birds than the forward observer (Figure 2). In addition, both observers detected fewer birds in distance bin 3 than in distance bin 4, contrary to expectation. Possibly, the requirement to scan across a 200 m field of view may induce both observers to unintentionally focus greater attention at the extremes (closest and farthest distances) of the transect than in the middle. Furthermore, the architecture of various fixed‐wing and helicopter aircraft can differ considerably, impacting the field of view for each observer. For example, bubble windows could reduce but not eliminate visibility issues associated with the presence of floats. Such unconscious idiosyncrasies of human observer behavior in combination with particular visibility issues specific to airframe design and window placement reinforce the fundamental requirement of any sampling design: that detection probability should be estimated in some fashion for improved inference, regardless of aircraft type used as a survey platform.

Further complications during sampling arise when there is responsive movement of animals between distance bins between detections of the same animal group by each observer. Such responsive movement can lead to (often positive) bias in estimates of abundance (Borchers, Marques, Gunnlaugsson, & Jupp, 2010; Conn & Alisauskas, 2018) from MRDS models, and can impede estimation of MRDS parameters that represent individual heterogeneity (Burt et al., 2014). Although MRDS models are preferable to MR or MRD models when assumptions are met, there was considerable evidence for responsive movement in our data set which led us to consider simpler MR and MRD models that did not include a probability mass function for the distance data. For comparison between MR and MRD models, we edited distance bin observations in favor of the front observer to eliminate any mismatches in this study. Clearly this was not ideal in practise, but permitted a test of past data collection methods without distances recorded, against an improved standard that included distance information. Our use of MRD compared to MR showed vast improvements of model fit to data. Further improvement could likely be realized by explicitly modeling movement and measurement error with unedited records, as done by Conn and Alisauskas (2018). In particular, their analysis suggested residual presence of individual heterogeneity above and beyond the simple MRD formulation we used here.

Researchers have several options for integrating counts with detection probability during such surveys from which to draw inference about density or abundance in a study area. For example, it may be advantageous to increase survey coverage by acquiring single observer count data from both sides of the aircraft (Hines et al., 2006). In this case, double‐observer protocols to estimate detection probability could be implemented for a portion of the survey, or during portions of flights that are “off‐transect” (Hines & Kay, 2006). For instance, one observer could re‐seat themselves behind the other observer, as done throughout our study. In an operational aerial survey, off‐transect double‐observer data for MRD estimates of detection probability could be gathered when the aircraft must suspend transect coverage to travel to and from refueling sites, for example. Then, abundance or density could be estimated in an ad hoc fashion with the simple canonical estimator, for example N = C/p*, or via model‐based analogs (e.g., Miller, Burt, Rexstad, & Thomas, 2013; Conn, Laake, & Johnson, 2012).

Our Arctic study area differed in an important way from the double‐observer methods used by Koneff et al. (2008) in a forested study area, farther south. The methods used in our study do not account for birds that are hidden from the field of view (e.g., by vegetation) within the sampled strip. We believe that such availability bias (Marsh & Sinclair, 1989) played no role for detecting birds that were most commonly observed on land in our study since vegetation was shorter than the birds under observation (Conkin & Alisauskas, 2013; Didiuk & Ferguson, 2005). However, detection probability was lowest for Loons and long‐tailed ducks, two species most often detected on water, and that tend to dive in response to approaching aircraft. King eider was another species most often detected on water, but their detection probabilities were similar to those of largely terrestrial cackling geese probably because their normal response was flight, rather than diving, in apparent evasive response to aircraft. Rock Ptarmigan also had lower detection probability, possibly due to the very cryptic plumage of females in particular.

We used relatively simple models for waterfowl detection data where species was a fixed effect on detection. Our study focussed on relatively common species, but to include rarer species, it would likely be necessary to borrow strength from an ensemble of species. For example, species could be treated as a random effect within a hierarchical modeling framework (see e.g., Sollman, Gardner, Williams, Gilbert, & Veit, 2016). For larger surveys, one may also wish to estimate species–habitat relationships (e.g., Conkin & Alisauskas, 2013), permitting prediction of abundance in unsurveyed locations (e.g., Miller et al., 2013, Sollman et al., 2016).

Our work suggested that past helicopter surveys that used only MR methods on fixed‐width transects without distance data (e.g., Hines & Kay, 2006; Hines et al., 2006) may have provided reasonably accurate inference about abundance. However, such tests between MR and MRD methods for estimating detection and bias should probably be done for other types of aircraft design and configuration that affect visibility. Data can be tractably gathered to model effects of different observers, responsive animal behavior, and errors in distance determinations (Conn & Alisauskas, 2018), but additional covariates that presumably affect detection probability, such as airspeed, deviations from altitude, cloud coverage, habitat type etc., could also be recorded to the extent possible.

Overall, because of the added benefits of apparent reductions in bias and especially improvement precision for some species, we urge that surveys of waterfowl or other wildlife from either fixed‐wing aircraft or helicopters consider distance information in conjunction with double‐observer methods. Although we found that our MR estimator which ignored distance data did not result in appreciably biased estimates, the markedly improved estimates of precision when using DS in conjunction with double‐observer methods could improve ability to test for differences in abundance or density among regions or between years. Modeling both spatial and temporal change in population abundance is a central focus behind decisions about population health. Improved precision of estimates when MRD (this study) or MRDS methods (Conn & Alisauskas, 2018) are used provide added incentive for considering their application to data from aerial survey. We join other authors (e.g., Laake, Dawson, & Hone, 2008) in recommending that wildlife survey planners routinely collect distance data when conducting double‐observer surveys, if permitted by the configuration of the survey platform used and the behavior and mean group size of the species studied.

## AUTHORS’ CONTRIBUTIONS

RA conceived the hypothesis and design for testing whether inclusion of distance information improves estimation of Arctic wildlife abundance over currently used standards of aerial survey for waterfowl. RA collected the data, PC conducted analyses, and both authors co‐wrote this paper.

## Data Availability

Data available from the Dryad Digital Repository: https://doi.org/10.5061/dryad.mp7h92r
